# Accurate targeted long-read DNA methylation and hydroxymethylation sequencing with TAPS

**DOI:** 10.1186/s13059-020-01969-6

**Published:** 2020-03-03

**Authors:** Yibin Liu, Jingfei Cheng, Paulina Siejka-Zielińska, Carika Weldon, Hannah Roberts, Maria Lopopolo, Andrea Magri, Valentina D’Arienzo, James M. Harris, Jane A. McKeating, Chun-Xiao Song

**Affiliations:** 1 Nuffield Department of Medicine, Ludwig Institute for Cancer Research, University of Oxford, Oxford, OX3 7FZ UK; 2 Nuffield Department of Medicine, Target Discovery Institute, University of Oxford, Oxford, OX3 7FZ UK; 3 Oxford Genomics Centre, Wellcome Centre for Human Genetics, University of Oxford, Oxford, OX3 7BN UK

**Keywords:** Long-read sequencing, DNA methylation, Epigenetic phasing, Bisulfite-free, 5-Methylcytosine

## Abstract

We present long-read Tet-assisted pyridine borane sequencing (lrTAPS) for targeted base-resolution sequencing of DNA methylation and hydroxymethylation in regions up to 10 kb from nanogram-level input. Compatible with both Oxford Nanopore and PacBio Single-Molecule Real-Time (SMRT) sequencing, lrTAPS detects methylation with accuracy comparable to short-read Illumina sequencing but with long-range epigenetic phasing. We applied lrTAPS to sequence difficult-to-map regions in mouse embryonic stem cells and to identify distinct methylation events in the integrated hepatitis B virus genome.

**Supplementary information:**

**Supplementary information** accompanies this paper at 10.1186/s13059-020-01969-6.

## Background

Recent advances in third-generation sequencing methods, including PacBio SMRT sequencing [1–3] and Oxford Nanopore sequencing [4], have enabled long-read and single-molecule sequencing that is distinct from the mainstream short-read Illumina sequencing. These newer sequencing platforms allow unambiguous mapping of repetitive and complex regions of the genome and provide unprecedented opportunities for detecting structural variants, phasing haplotypes, and assembling genomes [5, 6]. While Nanopore sequencing still has a high error rate (~ 10%), the latest SMRT sequencing provides accuracy similar to Illumina sequencing (99.8%) but with an average read length of 13.5 kilobase (kb) compared to ~ 0.3 kb with Illumina [3].

Long-read sequencing of DNA modifications, particularly the two abundant modifications—5-methylcytosine (5mC) and 5-hydroxymethylation (5hmC) [7, 8], is needed to obtain phased epigenomes that will enable new understanding of the functions of epigenetic modifications, for example allele-specific methylation in genomic imprinting [9] and heterogeneous cancer samples, and diagnosis of brain tumors [10]. Although the SMRT and Nanopore platforms can detect DNA modifications directly, there are major barriers to their application. SMRT sequencing can directly detect DNA modifications using polymerase kinetics information, but requires a minimum of 250× per strand coverage to detect 5mC [11], largely defeating the purpose of long-read sequencing. Several computational methods have been developed to detect base modifications directly from Oxford Nanopore sequencing [12–15]. However, these approaches require complicated training data from control DNA samples of known methylation status and sophisticated computational analysis, limiting their accuracy to determine 5mC. Moreover, both native SMRT and Oxford Nanopore DNA methylation sequencing require microgram levels of native, unamplified DNA as input. Since amplification will erase any modifications, the application of these techniques on low-input samples, such as clinical materials, is limited. On the other hand, conventional bisulfite sequencing (BS-seq), which yields the sum of 5mC and 5hmC, is intrinsically difficult with long-read sequencing due to severe DNA degradation caused by bisulfite treatment, which limits read length of SMRT-BS to ~ 1.5 kb [16].

Recently, we described Tet-assisted pyridine borane sequencing (TAPS) [17], a novel 5mC and 5hmC detection method that utilizes mild reactions based on ten-eleven translocation (TET) enzyme oxidation of 5mC and 5hmC to 5-carboxylcytosine (5caC) and subsequent pyridine borane reduction of 5caC to dihydrouracil (DHU). During PCR amplification, DHU is recognized as thymine, resulting in a 5mC/5hmC-to-T transition. Technically, TAPS also detects the two minor DNA modifications, 5-formylcytosine (5fC) and 5caC, although they are only present at vanishingly small amounts in the mammalian genome (less than 0.002% of total cytosine) [18]. In contrast to harsh bisulfite treatment, TAPS preserves long DNA molecules over 10 kb, which is vital for long-read sequencing. TAPS induction of the 5mC/5hmC-to-T base change simplifies methylation detection on both SMRT and Nanopore sequencing platforms: with SMRT, 5mC would be detected by standard fluorescent changes rather than polymerase kinetics, thereby eliminating the need for ultra-high coverage to enable true long-read 5mC sequencing; with Nanopore, TAPS allows 5mC to be sequenced as a normal base, avoiding the need for training data and complex analysis, and thereby improving detection accuracy.

## Results and discussion

**Fig. 1:**
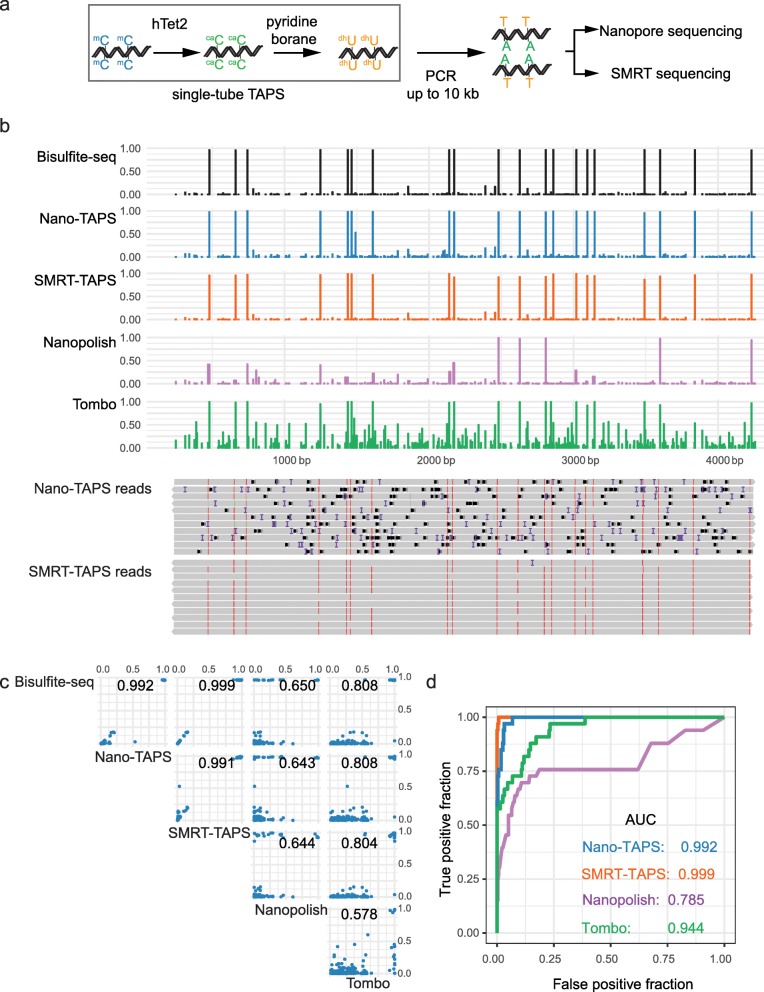
lrTAPS of 4 kb model DNA. **a** Schematic of lrTAPS for targeted long-read DNA methylation sequencing. **b** The upper panel (from top to bottom): methylation of a 4-kb model DNA obtained from bisulfite sequencing (short-read Illumina sequencing), Nano-TAPS, SMRT-TAPS, and native Nanopore methylation sequencing using Nanopolish or Tombo. The lower panel shows examples of individual long-reads from Nano-TAPS and SMRT-TAPS. The red bars indicate methylation. Black and purple bars indicate sequencing errors (deletions and insertions, respectively). **c** Scatter plots showing all pairwise correlations of all CpG sites among Nano-TAPS, SMRT-TAPS, native Nanopore methylation calling (Nanopolish and Tombo), and Bisulfite-seq, with correlation coefficient showing on top of each plot. **d** ROC curve and AUC comparing Nano-TAPS, SMRT-TAPS, and native Nanopore methylation sequencing (Nanopolish and Tombo), using DNA methylation from bisulfite sequencing (methylation level > 3% was designated as methylated) as the truth

To implement long-read TAPS (lrTAPS), we first developed a single-tube TAPS where the TET oxidation and pyridine borane reduction are performed in the same tube (Fig. 1a). This minimizes the loss of long DNA molecules during lrTAPS and allows for low DNA input. Furthermore, we used recombinant *E. coli*-expressed human TET2 (hTet2) instead of mammalian cell-expressed mouse Tet1 (mTet1) used in our previous study, which can be produced in high yield and at low cost. hTet2 retained comparable activity with mTet1 in the CpG context but showed ~ 10% lower efficiency in the CpH context than mTet1 (Additional file 1: Table S1). We used a 4-kb model DNA treated with HpaII methyltransferase, which methylates the internal cytosine residue in C-C-G-G sequences to C-5mC-G-G, while generating low-level off-target methylation in related sequences [19]. The methylation status of the 4 kb model DNA was determined by BS-seq using Illumina sequencing (Fig. 1b, top panel). We applied lrTAPS on the model DNA followed by long-range PCR amplification. The resulting amplicon was sequenced on both Oxford Nanopore and SMRT sequencing platforms (termed Nano-TAPS and SMRT-TAPS respectively), with methylation sites identified by CG-to-TG/CA substitutions compared to the reference sequence. Both Nano-TAPS and SMRT-TAPS successfully detected all of the methylated CCGG sites and most of the off-target sites showing a high agreement with BS-Seq data (Pearson correlation coefficient 0.992 and 0.999, respectively). SMRT-TAPS detected 5mC with only 3 passes in the single-molecular circular consensus sequence (CCS) mode and achieved higher accuracy than Nano-TAPS, consistent with the recent improvement in the accuracy of SMRT sequencing [3] (Fig. 1b,c). We also subjected the non-amplified TAPS-treated DNA, which contains DHU, to SMRT sequencing and found it stalls the polymerase used in the system (data not shown), suggesting DHU is incompatible with SMRT sequencing. When the native model DNA (i.e., without lrTAPS) was sequenced by Nanopore sequencing and methylation sites were called using Nanopolish [13] or Tombo [14] software, we noted a reduced agreement with BS-seq data (Pearson correlation coefficient 0.65 and 0.808, respectively, Fig. 1b,c). Receiver operating characteristic (ROC) analysis confirmed Nano-TAPS and SMRT-TAPS outperformed native Nanopore methylation sequencing with sensitivity and specificity comparable to Illumina sequencing (Fig. 1d).

To further assess the length limit of lrTAPS, we used HpaII methylated phage lambda DNA (48 kb). After TAPS conversion, the methylated DNA was PCR amplified to generate amplicons ranging from 3 to 10 kb (Additional file 1: Table S2). Complete lrTAPS conversion was confirmed by HpaII digestion (Additional file 1: Figure S1) and the longest 10 kb amplicon was sequenced by Oxford Nanopore and SMRT sequencing. For both platforms, we observed excellent agreement with BS-seq data in detecting DNA methylation (Pearson correlation coefficient 0.967 and 0.982, respectively. Additional file 1: Figure S2).

**Fig. 2:**
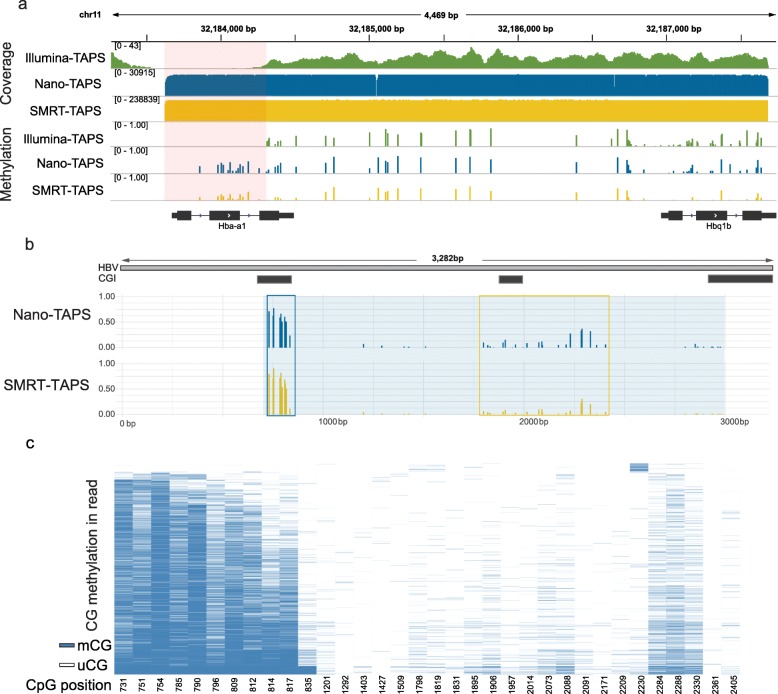
lrTAPS of a previously unmapped region in mESCs and integrated HBV DNA in Huh-1 cells. **a** Genome browser view of the methylation and coverage detected by Illumina-TAPS, Nano-TAPS, and SMRT-TAPS in Hba-a1 locus. The pink shaded area shows the gap which cannot be mapped with Illumina short-read sequencing. **b** CpG methylation of integrated HBV DNA in Huh-1 cells detected by Nano-TAPS and SMRT-TAPS. The blue shaded area shows the covered regions with lrTAPS. Regions of methylated CpGs are indicated by the blue/yellow boxes. **c** Heatmap showing integrated HBV DNA methylation in each SMRT read (34,755 reads were included). Reads were ranked by the average methylation in the first CpG Island. The blue bar indicates the methylated CpG (mCG) while white bar indicates unmethylated CpG (uCG). The number in the bottom indicates the relative position of CpG in the HBV reference genome

Long-read sequencing has been successfully applied to characterize difficult-to-map DNA and close gaps in human genome assemblies [4, 20]. Indeed, we noted gaps in our previously reported TAPS analysis of mouse embryonic stem cells (mESCs) determined by the Illumina sequencing (Illumina-TAPS) [17] (Fig. 2a). We applied the lrTAPS method to 50 ng of E14 mESCs genomic DNA and amplified a 4-kb region that spans a 500-bp gap previously identified on chromosome 11. Both Nano-TAPS and SMRT-TAPS detected methylated CpG sites in the gap, which contains *Hba-a1* (encoding hemoglobin alpha, adult chain 1), a previously unmappable gene that has an identical sequence to its homolog *Hba-a2* (encoding hemoglobin alpha, adult chain 2) (Fig. 2a and Additional file 1: Figure S3) [21]. Across the 4-kb region (outside of the gap), Nano-TAPS and SMRT-TAPS showed good correlation with Illumina-TAPS at CpG sites with sequencing depth > 8 (Pearson correlation coefficient 0.893 and 0.913, respectively. Additional file 1: Figure S4), confirming that lrTAPS provides comparable results to Illumina sequencing of biological samples. The differences are most likely explained by the relatively low coverage of Illumina-TAPS (average depth 17× in this region) compared to the high coverage targeted sequencing of Nano-TAPS (14,600×) and SMRT-TAPS (210,100×). This demonstrated the power of lrTAPS to provide accurate DNA methylation maps of previously inaccessible non-unique genomic regions.

To further evaluate the utility of lrTAPS analysis of biological samples, we applied this method to study hepatitis B virus (HBV) DNA methylation. HBV is a global health problem with more than 250 million people chronically infected and at least 880,000 deaths/year from liver diseases [22]. HBV replicates via a 3.2-kb episomal copy of its genome, known as covalently closed circular DNA (cccDNA), and gene transcription is regulated by DNA methylation and other epigenetic modifications [23, 24]. A linear form of HBV DNA can be generated during viral replication that can integrate into the host genome [25]; these integrated viral DNA fragments may contribute to carcinogenesis [26]. However, our understanding of the role DNA methylation plays in the HBV life cycle and associated pathogenesis is limited by the insensitivity of BS-seq or methylation-specific PCR to quantify the HBV DNA methylation status [27]. With lrTAPS, we show for the first time that HBV cccDNA in de novo infected HepG2-NTCP (HepG2 cells engineered to express sodium taurocholate co-transporting polypeptide (NTCP) which support the full HBV life cycle) is unmethylated (Additional file 1: Figure S5), consistent with active transcription and genesis of infectious particles [28]. In contrast, integrated copies of HBV DNA [29] in Huh-1 hepatoma cells are methylated at the predicted CpG islands (CGI) and gene body (Fig. 2b). Another major benefit of lrTAPS is the ability to phase long-range epigenetic variations at a single molecule level [30]. Indeed, further analysis of the methylation at the level of single long reads shows distinct methylation events on the HBV genome that are either correlated or anti-correlated over long distances, indicating heterogeneity of DNA methylation status among integrated HBV DNA (Fig. 2c and Additional file 1: Figure S6). Such feature could only be uncovered with the phased methylome delivered by long-read sequencing and is important for studying heterogeneous samples such as patient-derived material.

In summary, the lrTAPS approach enables accurate long-read DNA methylation and hydroxymethylation sequencing and could open many research possibilities by allowing accurate, simple, and cost-effective analysis of DNA methylation using nanogram quantities of input DNA. The long-range DNA methylation phasing delivered by lrTAPS, especially when realized with the more accurate SMRT sequencing, could provide great opportunities to study allele-specific methylation [9]. With a suitable long-range amplification method [31], this approach can also be applied in the future to give whole-genome long-read methylation profiles. Future development of modified lrTAPS [17] could potentially distinguish 5mC and 5hmC in long-read sequencing.

## Methods

### Preparation of model DNA

The 4-kb model DNA was prepared by PCR amplification of pNIC28-Bsa4 plasmid (Addgene, cat. no. 26103) and methylated by HpaII Methyltransferase (NEB, M0214S). Unmethylated lambda DNA (Promega) was also methylated by HpaII Methyltransferase for C^m^CGG methylation. Detailed protocol is provided in Additional file 1: Supplementary method 1.

### Cell culture and isolation of genomic DNA

E14 mESCs (gift from S. Kriaucionis) were cultured on gelatin-coated plates in DMEM (Invitrogen) supplemented with 15% FBS (Gibco), 2 mM l-glutamine (Gibco), 1% nonessential amino acids (Gibco), 1% penicillin/streptavidin (Gibco), 0.1 mM β-mercaptoethanol (Sigma), 1000 units ml^−1^ leukemia inhibitory factor (Millipore), 1 μM PD0325901 (Stemgent) and 3 μM CHIR99021 (Stemgent). Huh-1 and HepG2-NTCP cells [28] were maintained in Dulbecco’s modified Eagle’s medium (DMEM) supplemented with 10% FBS, 2 mM l-glutamine, 1 mM sodium pyruvate, 50 U/mL penicillin/streptomycin, and non-essential amino acids (Thermo Fisher Scientific). HBV ayw stocks were purified from HepAD38 producer cells as previously reported [28]. HepG2-NTCP cells were treated with 2.5% dimethyl sulphoxide (DMSO) for 3 days and inoculated with HBV at a multiplicity of infection of 200 in the presence of 4% polyethylene glycol 8000. After 18–20 h, the inoculation was removed by washing with PBS and cells cultured in the presence of 2.5% DMSO. Cultures were maintained at 37 °C and 5% CO_2_. For isolation of genomic DNA, cells were harvested by centrifugation for 5 min at 1000*g* and room temperature. DNA was extracted with Quick-DNA Plus kit (Zymo Research) according to the manufacturer’s protocol.

### Expression and purification of hTet2

Protein was expressed in *E. coli* BL21 (DE3) from pET28a plasmid encoding engineered hTet2 protein (1129–1936-Δ(1481–1843), deletion replaced by 15 amino acids GS-linker) with 6xHis-Flag-SUMO N-terminal tag [32]. Overnight small-scale bacteria culture was grown in LB medium supplemented with 50 μg/mL kanamycin at 37 °C and 200 rpm until OD600 was between 0.75 and 0.9. Then cultures were cooled down to room temperature and target protein expression was induced with 0.2 mM isopropyl-β-d-1-thiogalactopyranoside (IPTG). Cells were maintained for additional 18 h at 18 °C and 180 rpm. Subsequently, cells were harvested and re-suspended in the lysis buffer containing 20 mM HEPES pH = 7.4, 500 mM NaCl, 20 mM imidazole, 0.5 mM TCEP, and 1× cOMPLETE protease inhibitor cocktail. Cells were broken by sonication, and lysate was clarified by centrifugation for 1 h at 30000×*g* and 4 °C. Collected supernatant was loaded on Ni-NTA resins and hTet2 protein was eluted with buffer containing 50 mM HEPES pH = 7.4, 500 mM NaCl, 250 mM imidazole, and 0.5 mM TCEP. Collected fractions were then purified on HiLoad 16/60 Sdx 75 (50 mM HEPES pH = 7.5, 500 mM NaCl, 0.5 mM TCEP). Fractions containing hTet2 were then collected, concentrated, and buffer exchanged to the final buffer containing 50 mM HEPES pH = 7.5, 200 mM NaCl, 0.5 mM TCEP. Pure protein was mixed with glycerol (30% v/v) and aliquots were stored at − 80 °C.

### Long-read TAPS

One nanogram of model DNA or 50 ng of genomic DNA sample was incubated in a 20 μL reaction containing 50 mM HEPES buffer (pH 7.0), 100 μM ammonium iron(II) sulfate, 1 mM α-ketoglutarate, 2 mM ascorbic acid, 1 mM dithiothreitol, 100 mM NaCl, 1.2 mM ATP, and 4 μM hTet2 for 80 min at 30 °C. Then 0.8 U of Proteinase K (NEB) were added to the reaction and incubated at 50 °C for 1 h. After cooling down to room temperature, 6 μL of 3 M sodium acetate solution (pH = 4.3) and 3 μL of 10 M pyridine borane (Alfa Aesar) were added to the reaction mixture directly and incubated at 37 °C and 850 rpm in a ThermoMixer (Eppendorf) for 16 h. The reaction was purified with Zymo-IC column (Zymo Research) and Oligo Binding buffer (Zymo Research). The converted DNA was then amplified with LongAmp Hot Start Taq 2X Master Mix (NEB). The detailed protocol is described in Additional file 1: Supplementary method 2. Primer sequences are listed in Additional file 1: Table S2.

### Illumina-TAPS

Illumina-TAPS was done according to previous protocol [17] with minor changes. Fragmented and size-selected genomic DNA was ligated with sequencing adaptors and treated with same lrTAPS protocol above except additional purification with 1.8× Ampure XP beads before pyridine borane reaction. The detailed protocol is described in Additional file 1: Supplementary method 3.

### Restriction enzyme digestion assay

After PCR amplification, 50 ng of lrTAPS product was incubated with 4 units of HpaII restriction enzyme (NEB) in 1× CutSmart buffer (NEB) for 30 min at 37 °C and then visualized by 2% agarose gel electrophoresis. For successful lrTAPS conversion, the restriction site (CCGG) is lost due to the C-to-T transition and so the amplicon would remain intact. Genomic DNA samples were spiked-in with 0.5% of methylated 4 kb model DNA and lrTAPS conversion was validated by HpaII digestion assay on the model DNA.

### Bisulfite sequencing

A total of 50 ng of the methylated 4-kb model DNA or lambda-DNA was fragmented to by Covaris M220 instrument and size-selected to 200–400 bp using Ampure XP beads. End-repair and A-tailing reaction and ligation of methylated adapter (NextFlex) were prepared with KAPA HyperPlus kit (Kapa Biosystems) according to the manufacturer’s protocol. Subsequently, DNA underwent bisulfite conversion with EpiTect Bisulfite Kit (Qiagen) according to the manufacturer’s protocol. The final library was amplified with KAPA Hifi Uracil Plus Polymerase (Kapa Biosystems) for 6 cycles and cleaned up on 1× Ampure XP beads. The BS-seq library was paired-end 80 bp sequenced on a NextSeq 500 sequencer (Illumina).

### Nanopore sequencing

The 4-kb model DNA samples were sequenced on one MinION R9.4.1 RevD flow cell while mESCs and HBV samples were sequenced on one Flongle R9.4.1 flow cell. One microgram and 250 ng of each PCR product was used in the standard Native Barcoding genomic DNA (with EXP-NBD104, EXP-NBD114 and SQK-LSK109) protocol for the MinION and Flongle run, respectively. Reads were basecalled with guppy-2.3.5 flip flop model and demultiplexed with guppy_barcoder (v 2.3.5). Adapters in reads were trimmed with Porechop (v 0.2.3).

### SMRT sequencing

The 4-kb model DNA lrTAPS product was double-digested by BstAPI restriction enzyme (NEB) then ligated with modified SMRTbell adaptor (IDT, sequence 5′ to 3′ /5Phos/GTAGTCTCGCACAGATATCTCTCTCTTTTCCTCCTCCTCCGTTGTTGTTGTTGAGAGAGATATCTGTGCGAGACTACAGT, extra AGT overhang was added for the stick-end ligation) by Instant Sticky-end Ligase Master Mix (NEB). SMRTbell Template Prep Kit 1.0 (Pacbio) and standard 16-base barcode SMRTbell adaptors (IDT) were used for library preparation of lambda DNA, mESCs, and HBV samples. SMRTbell libraries were pooled in equimolar amounts for a total of 300 ng. For sequencing, the pooled SMRTbell library was bound with Sequel II Binding Kit 2.0, sequenced with Sequel II Sequencing Plate 2.0 using a 30-h movie with 1 h pre-extension time. Data were demultiplexed and CCS reads computed using the SMRT Analysis package (Pacific Biosciences) with minimum 3 passes and minimum predicted accuracy = Q20.

### Native methylation calling for Nanopore reads

C^m^CGG methylated 4-kb model DNA was used to evaluate the accuracy of native methylation calling algorithm for Nanopore sequencing. For Nanopolish (0.9.2) [13], nanopolish index was used to build an index mapping from basecalled reads and minimap2 2.16-r922 was used to align reads to reference with -x map-ont option. Methylated CpG was then detected with nanopolish call-methylation module, and calculate_methylation_frequency.py was used to calculate methylation. For Tombo (1.5) [14], tombo preprocess annotate_raw_with_fastqs was used to annotate read files with baseballs in FASTQ format. Tombo resquiggle was used to align raw signal to reference and tombo detect_modifications alternative_model was used to detect methylated CpG with --alternate-bases CpG --dna --multiprocess-region-size 1000 --processes 2 options.

### WGBS and TAPS data processing

For WGBS in the 4-kb model DNA or lambda-DNA, fastp [33] was used to preprocess the FASTQ files, and bismark (v0.22.0) [34] was used to map clean reads to reference. MarkDuplicates was used to remove PCR duplicates and bismark_methylation_extractor was used to extract methylation ratio. For TAPS in E14 mESCs, published data GSE112520 was processed as describe before [17]. Integrative Genomics Viewer (IGV) [35] was used to visualize individual long-read from Nano-TAPS and SMRT-TAPS and coverage/methylation in E14 mESCs and lambda-DNA.

### Methylation calling for lrTAPS

Long reads were mapped to reference genome using minimap2 (2.16-r922) [36] with -x map-ont option. For the 4-kb model DNA, from 2627 to 6911 of pNIC28-Bsa4 sequence was used as reference. It is worth noting that a 3-bp TAT deletion (position: 1996–1998) was detected in BS-seq, Nano-TAPS, and SMRT-TAPS and thus removed from the reference. For E14 mESCs, mm9 gnome was used as reference. For lambda DNA, the reference can be found under accession J02459. For HBV, the reference of HBV ayw strain can be found under accession number KX470733. The reads were filtered by length (as summarized in Additional file 1: Table S3), and methylated CpG was detected using a custom R script (mCG_lrtaps.r). Theoretically, the methylated CG was converted to TG or CA after TAPS, while un-methylated CG remained to be CG. The CG methylation ratio was thus calculated as the (TG + CA)/ (TG + CA + CG). In HBV genome specifically, (TG + CA + CG)/NN > 0.8 and non-TAPS control was used to distinguish methylated CpG from single nucleotide polymorphisms (SNP). To evaluate the performance of lrTAPS in 4 kb as compared to BS-seq, we performed receiver operating characteristic (ROC) analysis. CpG sites with a methylation level higher than 3% in bisulfite sequencing were designated as methylated, while a methylation level lower than this cut-off was designated as un-methylated. ROC was used to evaluate the performance of different methods with plotROC package (https://cran.r-project.org/web/packages/plotROC) [37], and calc_auc was used to compute the area under receiver (AUC).

### CGI detection in HBV

The CpG Islands in HBV genome are predicted with https://www.urogene.org/cgi-bin/methprimer/methprimer.cgi.

## Supplementary information

Additional file 1: **Figure S1.** Validation of lrTAPS by HpaII digestion. **Figure S2.** lrTAPS allows accurate detection of DNA methylation in regions up to 10 kb. **Figure S3.** Sequence alignment of Hba-a1 and Hba-a2. **Figure S4.** Scatter plot showing the correlation of methylation detected by Nano-TAPS, SMRT-TAPS, and Illumina-TAPS on the ~4 kb mESC genomic region shown in Fig. 2a. **Figure S5.** CpG methylation in HBV cccDNA isolated from infected HepG2-NTCP cells (6 days post-infection) detected by Nano-TAPS and SMRT-TAPS. **Figure S6.** Heatmap showing the Pearson’s correlation of methylation in each CpG sites measured by SMRT-TAPS in HBV integrated DNA in Huh-1 cells. **Table S1.** Comparison of hTet2 and mTet1CD activity by Illumina-TAPS. **Table S2.** Primers used for lrTAPS. Table S3. Sequencing and mapping statistics for long-read TAPS. Supplementary method 1. Preparation of model DNA and spike-in control. Supplementary method 2. Long-read TAPS. Supplementary method 3. Illumina-TAPS.

Additional file 2: Review history.

## Data Availability

All sequencing data are available in SRA under BioProject: PRJNA588716 [38]. The code used to process long-read TAPS data can be downloaded from https://github.com/jfeicheng92/lrTaps [39] and Zenodo [40]. The code is available under the MIT license.
