# Supplementary material for: Accurate targeted long-read DNA methylation and hydroxymethylation sequencing with TAPS
Source: Genome Biol. 2020 Mar 3;21:54. doi: 10.1186/s13059-020-01969-6 (PMC7053107; doi:10.1186/s13059-020-01969-6)
Supplement: Supplementary file 1 — Figure S1. Validation of lrTAPS by HpaII digestion. Figure S2. lrTAPS allows accurate detection of DNA methylation in regions up to 10 kb. Figure S3. Sequence alignment of Hba-a1 and Hba-a2. Figure S4. Scatter plot showing the correlation of methylation detected by Nano-TAPS, SMRT-TAPS, and Illumina-TAPS on the ~4 kb mESC genomic region shown in Fig. 2a. Figure S5. CpG methylation in HBV cccDNA isolated from infected HepG2-NTCP cells (6 days post-infection) detected by Nano-TAPS and SMRT-TAPS. Figure S6. Heatmap showing the Pearson’s correlation of methylation in each CpG sites measured by SMRT-TAPS in HBV integrated DNA in Huh-1 cells. Table S1. Comparison of hTet2 and mTet1CD activity by Illumina-TAPS. Table S2. Primers used for lrTAPS. Table S3. Sequencing and mapping statistics for long-read TAPS. Supplementary method 1. Preparation of model DNA and spike-in control. Supplementary method 2. Long-read TAPS. Supplementary method 3. Illumina-TAPS. [file 13059_2020_1969_MOESM1_ESM.docx]

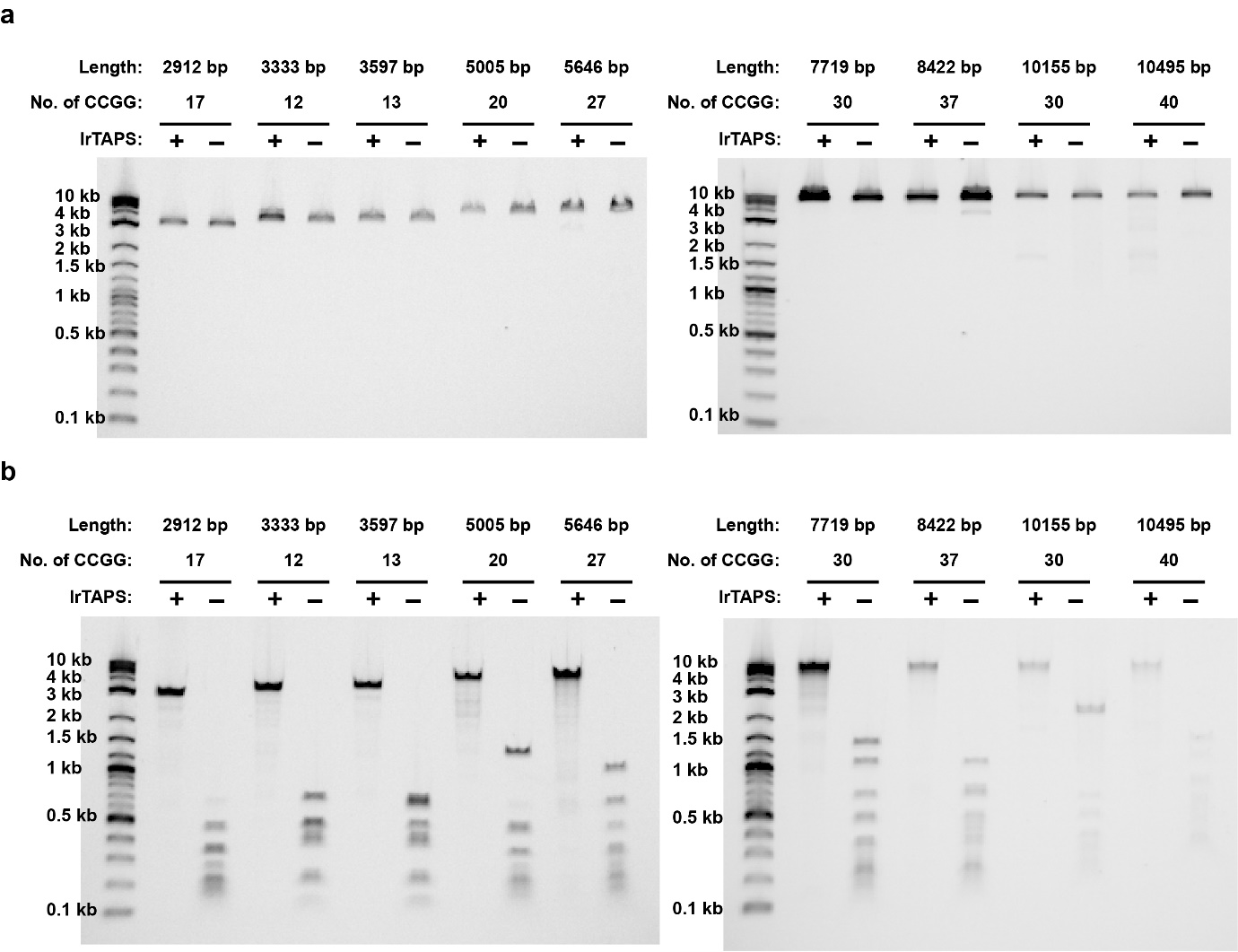


Figure S1. Validation of lrTAPS by HpaII digestion. Images of 1% Agarose gel analysis of PCR products of lambda model DNA, using primers listed in Supplementary Table 2 before (a) and after (b) HpaII digestion, and with (+) or without (-) lrTAPS conversion. The experiment was performed once.


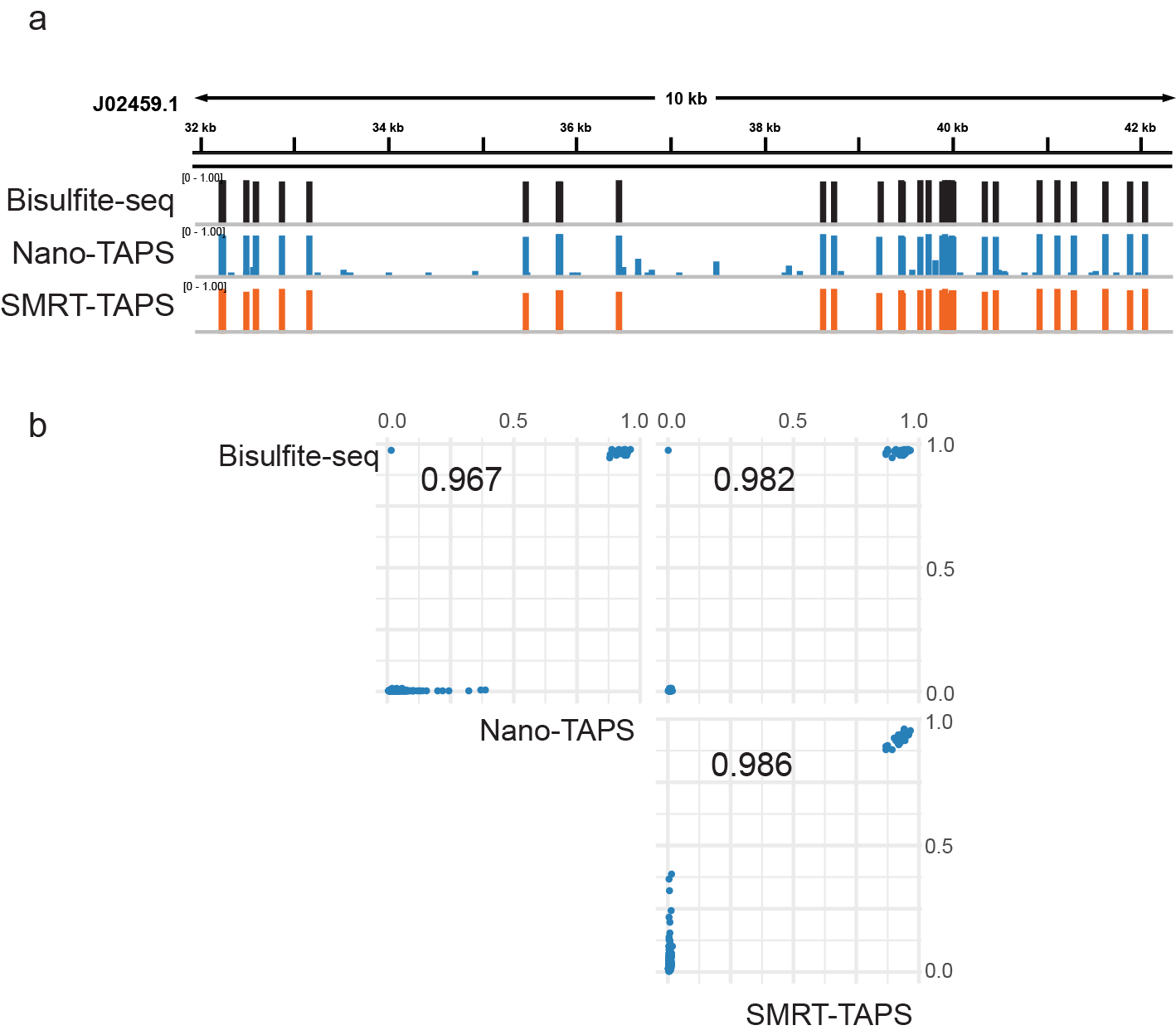


Figure S2. lrTAPS allows accurate detection of DNA methylation in regions up to 10 kb. (a) Integrative Genomics Viewer (IGV) snapshot depicting methylation in the 10 kb amplicon from lambda model DNA detected by BS-seq, Nano-TAPS and SMRT-TAPS. (b) Scatter plot showing all pairwise correlation among the methylation level detected by BS-seq, Nano-TAPS and SMRT-TAPS within all the CpG sites in the amplified region of the lambda, with correlation coefficient showing on top of each plot.


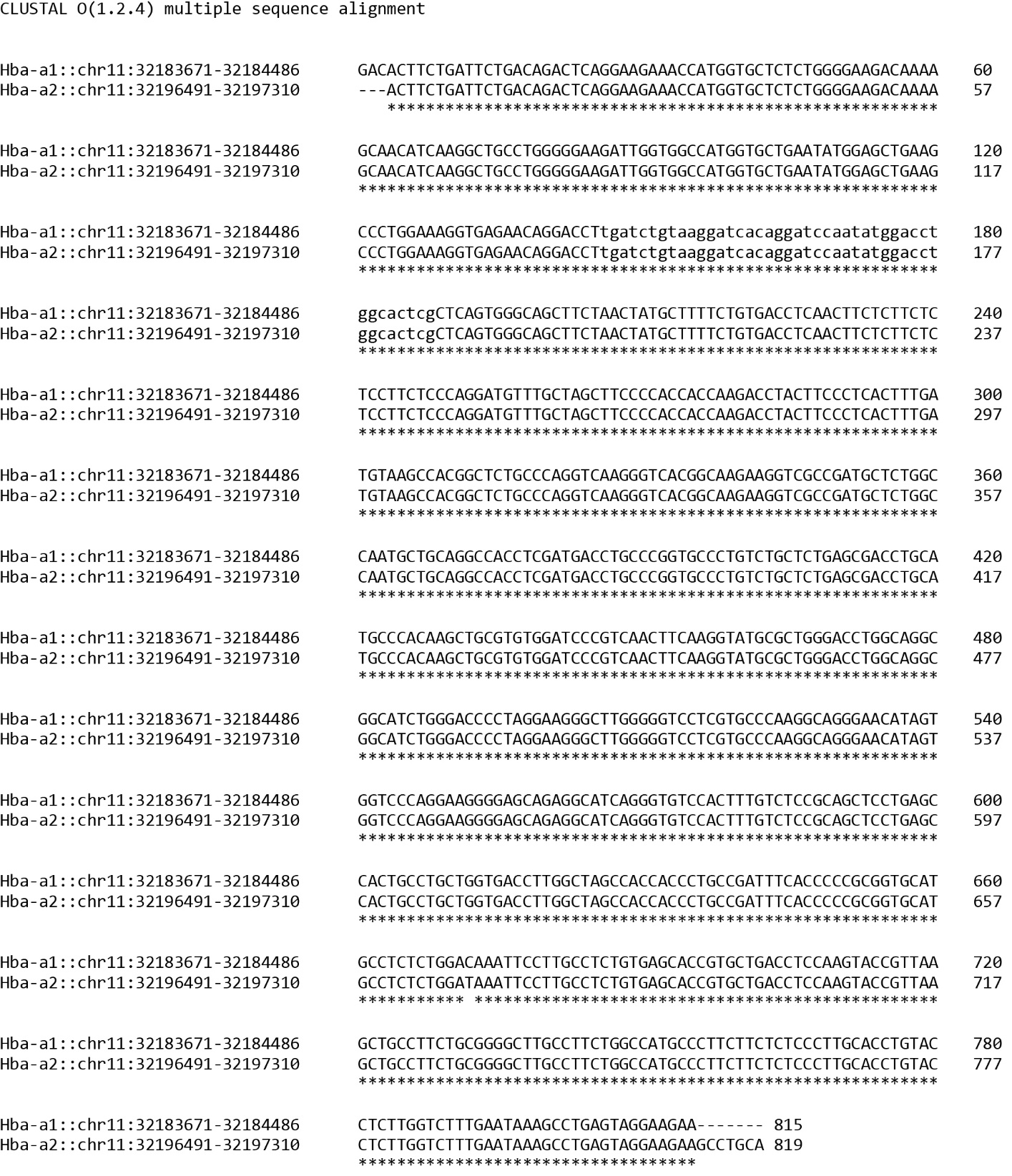


Figure S3. Sequence alignment of Hba-a1 and Hba-a2. Sequences of Hba-a1 and Hba-a2 were extracted based on mm9 genome and [annotation](http://hgdownload.soe.ucsc.edu/goldenPath/mm9/database/refGene.txt.gz).


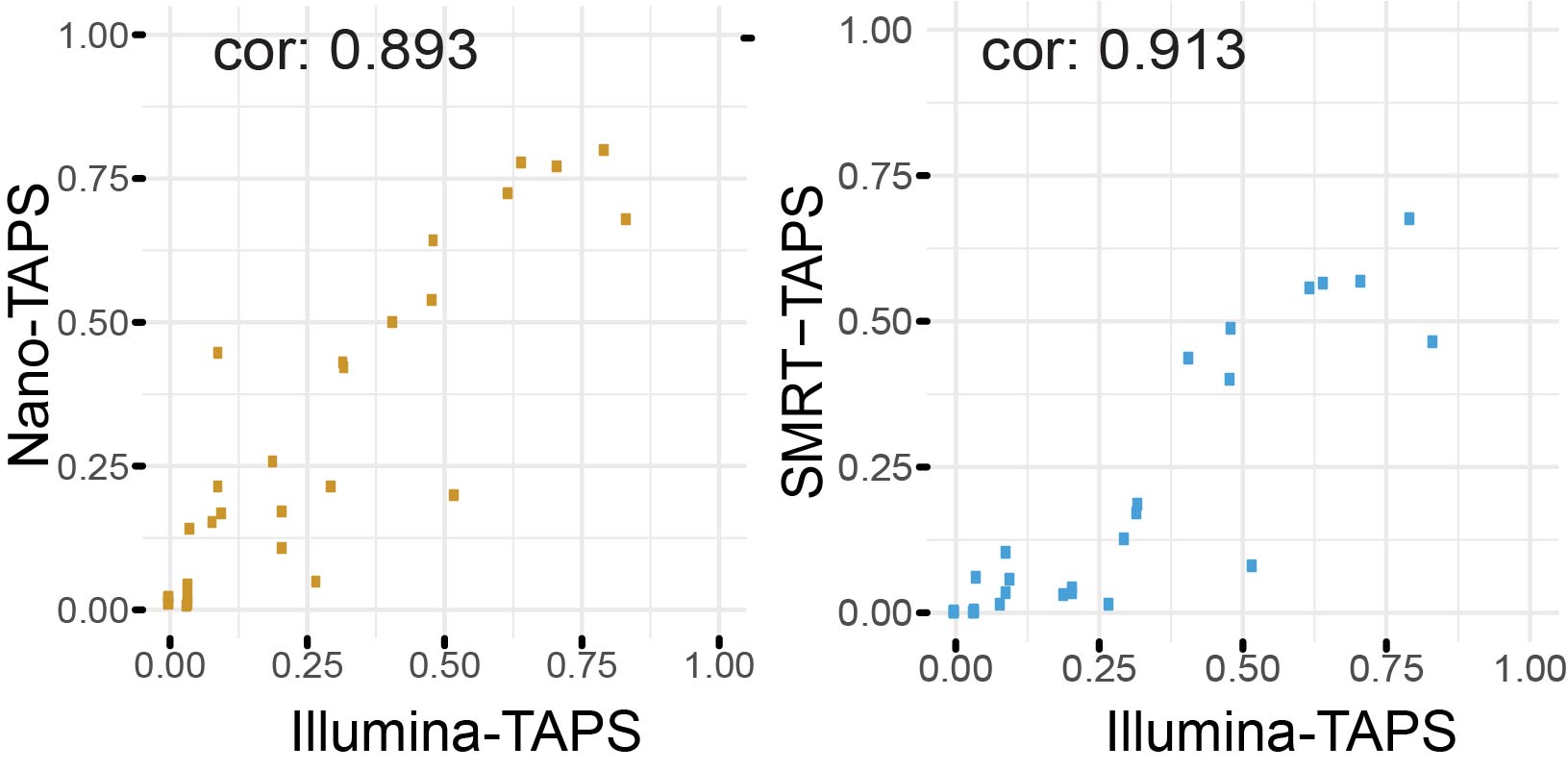


Figure S4. Scatter plot showing the correlation of methylation detected by Nano-TAPS, SMRT-TAPS, and Illumina-TAPS on the ~4 kb mESC genomic region shown in Fig. 2a, with correlation coefficient listed at the top of each plot. CG sites with Illumina sequencing depth >8x were selected for correlation analysis.


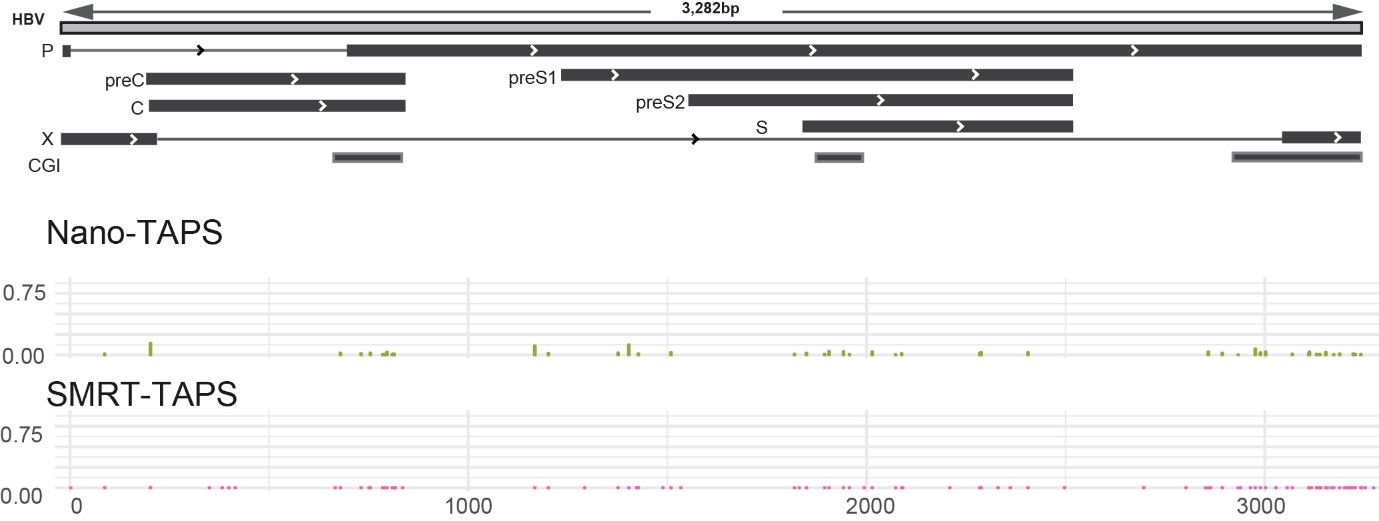


Figure S5. CpG methylation in HBV cccDNA isolated from infected HepG2-NTCP cells (6 days post-infection) detected by Nano-TAPS and SMRT-TAPS.


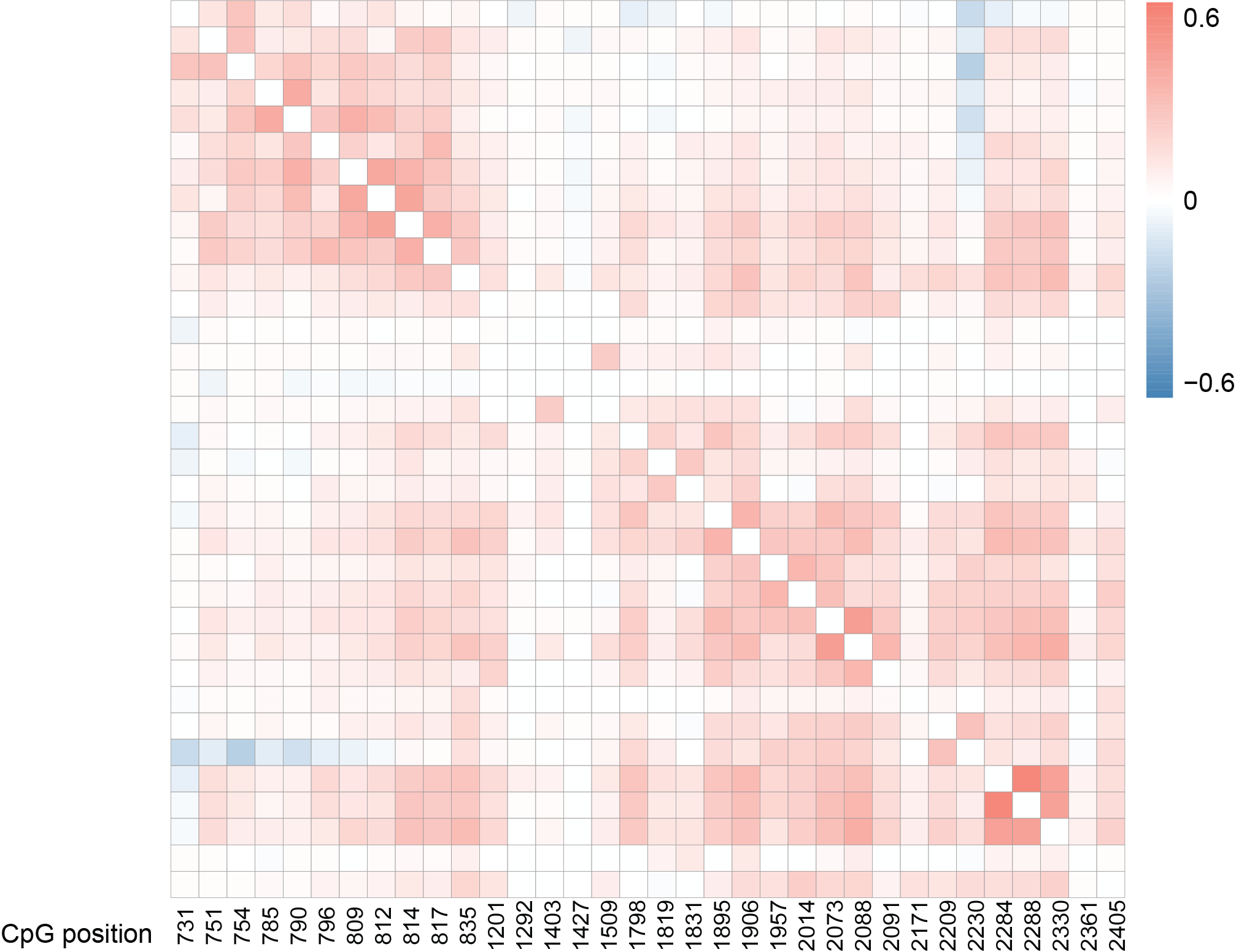


Figure S6. Heatmap showing the Pearson’s correlation of methylation in each CpG sites measured by SMRT-TAPS in HBV integrated DNA in Huh-1 cells. The methylation status for selected CpG sites in each read was designated to 0 for un-methylated CpG and 1 for methylated CpG as shown in Fig. 2c. The correlation of methylation status between these CpG sites is calculated with the cor function in R.

Table S1. Comparison of hTet2 and mTet1CD activity by Illumina-TAPS.

| TAPS conversion rate | hTet2 | mTet1CD |
| --- | --- | --- |
| mCpG | 97.3% | 97.3% |
| mCpH | 74.0% | 85.9% |
| hmCpG | 88.2% | 89.1% |
| hmCpH | 66.2% | 80.5% |
| Unmodified C | 0.19% | 0.23% |

An unmodified 2 kb amplicon was used to calculate conversion rate of unmodified C (false-positive rate). A synthetic oligonucleotide containing both a methylated and hydroxymethylated C surrounded by any other base (N5mCNN and N5hmCNN, respectively) was used to compare the conversion rate on 5mC and 5hmC in different sequence contexts. Details could be found in Nat Biotechnol. 2019; 37(4):424-9.

Table S2. Primers used for lrTAPS.

| Template | Primer | Sequence (5’ to 3’) | Note | Ta |
| --- | --- | --- | --- | --- |
| 4 kb model DNA | 4 kb-F | CATCGAGCATCAAATGAAACTGC | Nano-TAPS: 4 kb-F and 4 kb-R, amplicon size 4015 bp  SMRT-TAPS: 4 kb-F-BstAPI (to introduce BstAPI restriction enzyme site for stick-end ligation) and 4 kb-R | 60^o^C |
|  | 4 kb-R | ACGTTATACGATGTCGCAGAGT |  |  |
|  | 4 kb-F-BstAPI | ATCAGGTGGCACACTCTATCTCGGAAGCAGACTCTGCCATCGAGCATCAAATGAAACTGC |  |  |
| Lambda DNA | Lambda-F1 | CTTCGGCCTGTGTCAGTTCT | Combinations of primers for amplicons with different length:    F3R1-2932 bp; F5R3-3333 bp; F4R3-3597 bp; F2R1-5005 bp; F3R2-5646 bp; F2R2-7719 bp; F3R3-8422 bp; F1R1-10155 bp; F2R3-10495 bp | 60^o^C |
|  | Lambda-F2 | AACGTCTCTTCAGGCCACTG |  |  |
|  | Lambda-F3 | ATCGCACCATCAGCCAGAAA |  |  |
|  | Lambda-F4 | GGTGTGGCAAAGCTTGAAGG |  |  |
|  | Lambda-F5 | CTTACCCAACCCACCTGGTC |  |  |
|  | Lambda-R1 | CGGATATCCCACAGGTGAGC |  |  |
|  | Lambda-R2 | GCTCAGTTTGGGTTGTGCTG |  |  |
|  | Lambda-R3 | CCATGCGCTTGCTCTTCATC |  |  |
| mESC gDNA | Chr11-F | ACGCCCTTGGAGGGCATA | Expected amplicon size 4053 bp | 60^o^C |
|  | Chr11-R | AGGGCATGGGTGGAGACTAT |  |  |
|  | Chr13-F | TTAGCTGCACCTTTGTGCTT | Expected amplicon size 4407 bp | 60^o^C |
|  | Chr13-R | TTGCACCCTGTCTGCAATCT |  |  |
| HBV cccDNA | ApaLI-F | CACGTCGCATGGAGACCAC | Expected amplicon size ~3.2 kb | 60^o^C |
|  | ApaLI-R | CCGGCAGATGAGAAGGaACAG |  |  |
| Huh-1 HBV DNA | HepB8F | CTTATAGACCACCAAATGCCCCTA | Expected amplicon size ~2.1 kb | 58^o^C |
|  | HepB22R | CAAAACAAGCGGCTAGGAGTTC |  |  |

Table S3. Sequencing and mapping statistics for long-read TAPS.

| Sample | # Raw reads | # Raw bases | Average length | # Mapped reads | length filter criteria(bp) | # reads after filtering |
| --- | --- | --- | --- | --- | --- | --- |
| nanopore_4kb.ccgg_meth.noTAPS | 26,845 | 111,775,211 | 4,164 | 26,842 | 3,500 | 25,377 |
| nanopore_4kb.ccgg_meth.TAPS | 33,473 | 96,610,038 | 2,886 | 33,459 | 3,500 | 20,970 |
| pacbio_4kb.ccgg_meth.TAPS | 14,614 | 58,127,652 | 3,978 | 14,608 | 3,500 | 6,003 |
| nanopore_lambda.ccgg_meth.TAPS | 22,043 | 116,830,121 | 5,300 | 21,908 | 8,000 | 7,331 |
| pacbio_lambda.ccgg_meth.TAPS | 244,319 | 2,032,755,069 | 8,320 | 244,317 | 8,000 | 176,045 |
| nanopore_mESC_chr11.32183629_32187681.TAPS | 33,213 | 128,786,174 | 3,878 | 33,128 | 3,000 | 31,036 |
| nanopore_mESC_chr13.101123190_101127596.TAPS | 18,449 | 74,283,198 | 4,026 | 18,400 | 3,000 | 16,355 |
| pacbio_mESC_chr11.32183629_32187681.TAPS | 240,368 | 979,074,104 | 4,073 | 240,363 | 3,000 | 238,840 |
| pacbio_mESC_chr13.101123190_101127596.TAPS | 102,399 | 448,827,358 | 4,383 | 102,396 | 3,000 | 100,453 |
| nanopore_hbv_cccDNA.TAPS | 23,472 | 70,973,805 | 3,024 | 23,339 | 3,000 | 21,479 |
| pacbio_hbv_cccDNA.TAPS | 80,436 | 259,298,934 | 3,224 | 80,418 | 3,000 | 79,074 |
| nanopore_hbv_huh1.TAPS | 28,688 | 57,271,499 | 1,996 | 26,848 | 2,000 | 23,127 |
| pacbio_hbv_huh1.TAPS | 34,973 | 78,186,883 | 2,236 | 34,895 | 2,000 | 34,756 |

Supplementary method 1. Preparation of model DNA and spike-in control.

4 kb model DNA was prepared by PCR amplification of pNIC28-Bsa4 plasmid (Addgene, cat. no. 26103) and the reaction contained 1 ng DNA template, 0.5 μM primers and 1X Phusion High-Fidelity PCR Master Mix with HF Buffer (Thermo Scientific, F531S). Primers sequences are as following: forward primer 5’-ACTGGAACAACACTCAACCCTA-3’ and reverse primer 5’-AGGGTGGTGAATGTGAAACC-3’ and PCR conditions are:

| STEP | TEMP | TIME |
| --- | --- | --- |
| Initial Denaturation | 98°C | 30 seconds |
| 25 Cycles | 98°C 62°C 72°C | 10 seconds 15 seconds 63 seconds |
| Final Extension | 72°C | 10 minutes |
| Hold | 4-10°C |  |

The PCR product was purified by Zymo-IC column (Zymo Research) with Buffer PB (Qiagen) and the concentration was measured with Qubit dsDNA HS Assay Kit (ThermoFisher, Q32854) and purity checked by 1% agarose gel electrophoresis. The purified amplicon was methylated by HpaII Methyltransferase (NEB, M0214S) for 2 h at 37°C in a 50 μL reaction as following:

|  | 50 µL | Final |
| --- | --- | --- |
| Nuclease-free Water | To 50 µL |  |
| 10X CutSmart buffer | 5 µL | 1X |
| SAM (32 mM) | 1 µL | 0.64 mM |
| DNA | Various | 1 µg |
| HpaII methyltransferase (4 U/µL) | 2.5 µL | 0.2 U/µL |

After 2 h, 1.25 µL of HpaII methyltransferase and 1 µL of SAM were added to the reaction and incubated at 37°C for another 2 h. The reaction was purified with 1X Ampure XP beads (Beckman Coulter, A63881) according to the manufacturer’s protocol. DNA methylation was validated by HpaII digestion and 50 ng of methylated and unmethylated DNA digested in a 10 μL reaction with 2 U of HpaII restriction endonuclease (NEB) in 1X CutSmart buffer (NEB) for 1 h at 37°C. Unmethylated lambda DNA (Promega) was methylated with the same protocol above for C^m^CGG methylation.

Supplementary method 2. Long-read TAPS.

1. Assembly 20 μL reaction in a 1.5 mL DNA LoBind Tubes (Eppendorf) on ice as following:

|  | Volume | Final |
| --- | --- | --- |
| Purified DNA | Various | Up to 100 ng |
| hTet2 buffer | 6 μL | 1X |
| 1.5 mM Fe | 1.4 μL | 100 µM |
| H_2_O | To 20 μL |  |
| hTet2 | ~2 μL | 4 μM |

hTet2 buffer: 167 mM HEPES pH = 7.0, 333 mM NaCl, 3.3 mM α-KG, 6.67 mM L-AA, 4 mM ATP, 8.33 mM DTT. 1.5 mM Fe: dissolve 14.7 mg Fe(NH_4_)_2_(SO_4_)_2_ 6H_2_O to 1 mL of water, then make a 24-fold dilution. Optional: add 0.5%-1% methylated lambda DNA to the purified DNA sample as spike-in control for conversion test.

1. Incubate reaction at 30°C for 80 min. Add 1 µL of Proteinase K (NEB, P8107S, 0.8 U/ µL) to the oxidation reaction and incubate for 1 h at 50°C.
2. Prepare 3 M sodium acetate buffer solution (NaAc, pH = 4.3) by adjusting pH of 4 M acetic acid with 5 M sodium hydroxide solution. Add 6 µL of 3 M NaAc and 3 µL of pyridine borane (Alfa Aesar, ~10 M) to the 21 µL DNA sample (final 30 µL reaction contains 600 mM NaAc and 1 M pyridine borane). Incubate at 37°C and 850 rpm in a ThermoMixer (Eppendorf) for 16 h.
3. Purify the reaction on Zymo-IC column (Zymo Research) with Oligo binding buffer (Zymo Research) according to the manufacturer’s protocol. Elute in 20 µL of water. Optional: check the concentration of converted DNA with Qubit dsDNA HS Assay Kit and calculate recovery. Typical recovery yield is 40% to 80%.
4. Amplify the converted DNA with LongAmp Hot Start Taq 2X Master Mix (NEB) and primers for target regions (**Table S2**) using following PCR program:

| STEP | TEMP | TIME |
| --- | --- | --- |
| Initial Denaturation | 94°C | 30 seconds |
| 25 to 35 Cycles | 94°C Ta 65°C | 10 seconds 15 seconds 50 seconds per kb |
| Final Extension | 65°C | 10 minutes |
| Hold | 4-10°C |  |

1. Purify the PCR product with Ampure XP beads according to the manufacturer’s protocol. Measure the concentration with Qubit™ dsDNA HS Assay Kit and check the quality and purity on 1% agarose gel.

Supplementary method 3. Illumina-TAPS.

1. Fragment genomic DNA with Covaris M220 instrument to desired length (e.g. ~200 bp) in EB buffer and size-select with AMPure XP beads (e.g. 0.55X – 1X for 200 bp–400 bp). Necessary control spike-ins should be added before or after the fragmentation, depending on the length.
2. Use 100 ng of fragmented and size selected DNA from above for end-repair and A-tailing reaction with KAPA Hyper kit. For the ligation step, use following pre-annealed adapters instead of the standard KAPA index adapters:

5’-ACACTCTTTCCCTACACGACGCTCTTCCGATCT-3’ (IDT, HPLC purified)

5’-/5Phos/GATCGGAAGAGCACACGTCT-3’ (IDT, HPLC purified)

Purify Ligated DNA with 0.88X AMPure XP beads and elute in 20 μL of nuclease-free water.

1. Assembly 50 μL hTet2 oxidation reaction on ice as following:

|  | Volume | Final |
| --- | --- | --- |
| Purified DNA | 20 μL |  |
| hTet2 buffer | 15 μL | 1X |
| 1.5 mM Fe | 3.3 μL | 100 µM |
| H_2_O | To 50 μL |  |
| hTet2 | ~5 μL | 4 μM |

1. Incubate the reaction at 30°C for 80 min. Add 2 µL of Proteinase K (NEB, P8107S, 0.8 U/ µL) to the oxidation reaction and incubate for 1 h at 50°C.
2. Purify the oxidation reaction with 1.8X AMPure XP beads and elute in 20 μL of nuclease-free water. Repeat step 3) and 4) to achieve more complete oxidation. Elute double-oxidized DNA in 35 μL of nuclease-free water.
3. Add 10 µL of 3 M NaAc pH=4.3 and 5 µL of pyridine borane to the 35 µL DNA sample. Incubate at 37°C and 850 rpm in a ThermoMixer (Eppendorf) for 16 h.
4. Purify the reaction on Zymo-IC column (Zymo Research) with Oligo binding buffer (Zymo Research). Elute in 15 µL of water and amplify the converted DNA in 50 µL PCR reaction with KAPA HiFi HotStart Uracil+ ReadyMix (KAPA) and indexed primers in NEBNext Multiplex Oligos for Illumina kit (NEB). The PCR program is as following:

| STEP | TEMP | TIME |
| --- | --- | --- |
| Initial Denaturation | 98°C | 45 seconds |
| 4 Cycles | 98°C 60°C 72°C | 15 seconds 30 seconds 30 seconds |
| Final Extension | 72°C | 1 minute |
| Hold | 4°C |  |

1. Purify the PCR product with 1X Ampure XP beads. Measure the concentration with Qubit dsDNA HS Assay Kit (Invitrogen) and check the library on 2% agarose gel.
