# Supplementary material for: Accurate targeted long-read DNA methylation and hydroxymethylation sequencing with TAPS
Source: Genome Biol. 2020 Mar 3;21:54. doi: 10.1186/s13059-020-01969-6 (PMC7053107; doi:10.1186/s13059-020-01969-6)
Supplement: Supplementary file 2 — Review history. [file 13059_2020_1969_MOESM2_ESM.docx]

**First round**

**Reviewer 1**

The manuscript is straightforward and the data are clear, but the amount of information presented is minimal. The average reader would wish to know a good deal more about this exciting new application of TAPS, and a few points need to be clarified and strengthened.

1) The authors need to emphasize, in the title, abstract and all over the manuscript, that TAPS-seq will identify not only 5mC, but also 5hmC and the two other modified Cs (5fC and 5caC, which admittedly are present at vanishingly small amounts). This is because the initial treatment with TET will presumably also convert 5hmC and 5fC to 5caC. In fact the manuscript needs to incorporate information on how efficiently their recombinant E. coli-expressed human TET2 converts 5mC and 5hmC to 5caC. I understand that the authors are trying to compare TAPS with bisulfite sequencing, but it is widely accepted now that bisulfite sequencing yields the sum of 5mC and 5hmC. Perhaps "DNA methylation" could be replaced by "DNA cytosine modification" in the title, abstract and elsewhere?

2) Can the authors could show the effect of DHU on the kinetics (interpulse duration) of PacBio's DNA polymerase in non-amplified TABS-treated DNA. At a minimum, does DHU stall the polymerase? While this is not a major point, it is germane to the topic of the manuscript and the information would be useful to readers.

3) Likewise, a more detailed protocol for Illumina-TAPS and library preparation would be useful to the reader.

4) Fig 1c, S2b and other supplementary figures are missing the scale on the X- and Y-axes.

5) In Fig. S2, the authors used lambda DNA, therefore, all CCGG motifs should be methylated (i.e. all points should be at the top-right corner of the corresponding plots). Why then are some points at the lower left of the graph (i.e. read as demethylated) for both BS-based or TAPS-based strategies? If this is a problem with the HpaII methyltransferase treatment the authors need to present the efficiency of CCGG methylation by this enzyme. Alternatively, if the plot considers all cytosines or all CpG sequences in the DNA, this should be stated.

6) Fig. 2a compares amplicon-based NANO and SMRT TAPS sequencing against data from genome-wide Illumina bisulfite sequencing. Can the authors perform targeted sequencing for this region using the Illumina platform? This would provide a less biased comparison with regard to coverage.

7) The ROC threshold settings in Figure 1d are unclear and should be further described.

8) In Figure S6, the authors refer to concordant and discordant methylation but there is no information on how the authors calculated the correlation. Please describe in detail.

9) The authors describe the source of the pyridine borane as "Sigma" in the methods section but as "Alfa Aesar" in the supplementary material. Which is correct? If both were used, a comparison of the results should be shown so that readers who try to repeat the experiments will know which works better or if they are equivalent.

**Reviewer 2**

In this manuscript, Song and colleagues present a new methodology (lrTAPS) for targeted base-resolution sequencing of DNA methylation up to 10 kb from nanogram-level input. Overall the method is well developed, which was tested in different systems in the manuscript. This method could potentially be very useful to the field. The manuscript is well written. Below are some of my minor comments:

1. Could the presented approach distinguish 5hmC from 5mC? Based on Figure 1a, it seems that it would not. If so, how would the authors distinguish them?

2. Does hTet2 have any preference for CpG or non-CpG sites?

3. The authors demonstrated that 50 ng genomic DNA is sufficient for the presented method. Have they tried any amount lower than 50 ng?

**Response to reviewers**

**Reviewer 1**

GBIO-D-19-01886, Y Liu et al., Accurate targeted long-read DNA methylation sequencing with TAPS The manuscript is straightforward and the data are clear, but the amount of information presented is minimal. The average reader would wish to know a good deal more about this exciting new application of TAPS, and a few points need to be clarified and strengthened.

1) The authors need to emphasize, in the title, abstract and all over the manuscript, that TAPS-seq will identify not only 5mC, but also 5hmC and the two other modified Cs (5fC and 5caC, which admittedly are present at vanishingly small amounts). This is because the initial treatment with TET will presumably also convert 5hmC and 5fC to 5caC. In fact the manuscript needs to incorporate information on how efficiently their recombinant E. coli-expressed human TET2 converts 5mC and 5hmC to 5caC. I understand that the authors are trying to compare TAPS with bisulfite sequencing, but it is widely accepted now that bisulfite sequencing yields the sum of 5mC and 5hmC. Perhaps "DNA methylation" could be replaced by "DNA cytosine modification" in the title, abstract and elsewhere?

We appreciate the reviewer’s comment and have changed the title of the manuscript to “Accurate targeted long-read DNA methylation and hydroxymethylation sequencing with TAPS”, which is consistent with our previous TAPS publication. We also made changes in the abstract and main text accordingly. We further mentioned in the main text “Technically, TAPS also detects the two minor DNA modifications, 5-formylcytosine (5fC) and 5caC, although they are only present at vanishingly small amounts in the mammalian genome (less than 0.002% of total cytosine)”.

To demonstrate the efficiency of the E. coli-expressed hTET2 in TAPS, we further conducted Illumina sequencing with 5mC and 5hmC control DNA, which showed comparable efficiency in converting 5mC and 5hmC with the mammalian cell-expressed mTet1 used in our original TAPS paper:

| TAPS conversion rate | hTet2 | mTet1CD |
| --- | --- | --- |
| mCpG | 97.3% | 97.3% |
| mCpH | 74.0% | 85.9% |
| hmCpG | 88.2% | 89.1% |
| hmCpH | 66.2% | 80.5% |
| Unmodified C | 0.19% | 0.23% |

We have added this data to the supporting information, and this could also answer the question 2 from the reviewer #2. The advantages of hTET2 are the easy production, high yield, and low cost. We have provided detailed protocol for hTET2 expression and purification, which could further aid other labs to adopt the new technology.

2) Can the authors could show the effect of DHU on the kinetics (interpulse duration) of PacBio's DNA polymerase in non-amplified TABS-treated DNA. At a minimum, does DHU stall the polymerase? While this is not a major point, it is germane to the topic of the manuscript and the information would be useful to readers.

We appreciate the reviewer’s suggestion and conducted SMRT sequencing with non-amplified TAPS-treated 4 kb model DNA, which contains DHU, and we found it stalls the polymerase used in SMRT, suggesting DHU is incompatible with SMRT. We have added the result to this manuscript.

3) Likewise, a more detailed protocol for Illumina-TAPS and library preparation would be useful to the reader.

We appreciate the reviewer’s suggestion and have added a more detailed step-by-step protocol for Illumina-TAPS including library preparation (new Supplementary method 3).

4) Fig 1c, S2b and other supplementary figures are missing the scale on the X- and Y-axes.

We have added the scale for all figures.

5) In Fig. S2, the authors used lambda DNA, therefore, all CCGG motifs should be methylated (i.e. all points should be at the top-right corner of the corresponding plots). Why then are some points at the lower left of the graph (i.e. read as demethylated) for both BS-based or TAPS-based strategies? If this is a problem with the HpaII methyltransferase treatment the authors need to present the efficiency of CCGG methylation by this enzyme. Alternatively, if the plot considers all cytosines or all CpG sequences in the DNA, this should be stated.

The plot considers all CpG sites in the amplified regions of the lambda. There are some low-level but non-zero methylation events (lower left quadrant), which is due to the off-target activity of HpaII. We mentioned this in the main text and have added this statement in the legend.

6) Fig. 2a compares amplicon-based NANO and SMRT TAPS sequencing against data from genome-wide Illumina bisulfite sequencing. Can the authors perform targeted sequencing for this region using the Illumina platform? This would provide a less biased comparison with regard to coverage.

Actually in Fig. 2a we compared Nano-TAPS and SMRT-TAPS against genome-wide Illumina-TAPS, not bisulfite sequencing. One of exciting advantages of long-read sequencing is the ability to distinguish a gene of interest from its homolog, which is quite difficult for Illumina sequencing due to the inherent limitation of short read-length. Therefore we designed the experiment in Fig. 2a to demonstrate that lrTAPS could detect DNA methylation in a previously unmappable gene *Hba-a1* due to the identical sequence to its homolog *Hba-a2* and the detection is accurate and quantitative by comparing with Illumina-TAPS data outside of gap. When we increase the coverage cut-off of genome-wide Illumina-TAPS, we observed higher correlation with lrTAPS, which supports our statement on the coverage difference:

| coverage | # CpG sites | cor(Nanopore) | cor(Pacbio) |
| --- | --- | --- | --- |
| 2 | 61 | 0.819 | 0.816 |
| 4 | 60 | 0.854 | 0.863 |
| 6 | 56 | 0.854 | 0.859 |
| 8 | 38 | 0.893 | 0.913 |
| 10 | 29 | 0.893 | 0.904 |
| 12 | 21 | 0.903 | 0.889 |
| 14 | 6 | 0.963 | 0.967 |

We appreciate the reviewer’s concern about biased comparison due to the different coverage. However, we think this may not be the focus for Fig. 2a. Even if we did targeted sequencing for this region using Illumina platform by sequencing fragmented lrTAPS PCR product, it would only improve the correlation with lrTAPS and would not change the conclusion.

7) The ROC threshold settings in Figure 1d are unclear and should be further described.

We have added the detail of ROC threshold setting in the legend for Figure 1d and methods section. CpG sites with methylation level higher than 3% in bisulfite sequencing was designated as methylated, while methylation level lower than this cut-off was designated as un-methylated. ROC was used to evaluate the performance of different method with plotROC package, and calc_auc was used to compute the area under receiver (AUC).

8) In Figure S6, the authors refer to concordant and discordant methylation but there is no information on how the authors calculated the correlation. Please describe in detail.

We thank the reviewer for the advice and have added the method detail for calculated the correlation in the legend and methods section. The methylation status for selected CpG sites in each read was designated to 0 for un-methylated CpG and 1 for methylated CpG as shown in figure 2c. The correlation of methylation status between these CpG sites is calculated with the cor function in R.

9) The authors describe the source of the pyridine borane as "Sigma" in the methods section but as "Alfa Aesar" in the supplementary material. Which is correct? If both were used, a comparison of the results should be shown so that readers who try to repeat the experiments will know which works better or if they are equivalent.

We thank the reviewer for pointing out the mistake in our manuscript on the source of the pyridine borane, which has been corrected to "Alfa Aesar" in the methods section. In this manuscript, we used pyridine borane only from Alfa Aesar for lrTAPS, which is consistent with our previous Illumina-TAPS.

**Reviewer 2**

In this manuscript, Song and colleagues present a new methodology (lrTAPS) for targeted base-resolution sequencing of DNA methylation up to 10 kb from nanogram-level input. Overall the method is well developed, which was tested in different systems in the manuscript. This method could potentially be very useful to the field. The manuscript is well written. Below are some of my minor comments:

1. Could the presented approach distinguish 5hmC from 5mC? Based on Figure 1a, it seems that it would not. If so, how would the authors distinguish them?

The presented lrTAPS does not distinguish 5hmC from 5mC. We have changed the title and text to reflect this as suggested by both reviewers. In our original TAPS paper, we described two modified approaches, TAPSβ and CAPS, which can distinguish 5mC and 5hmC. It is our ongoing work to demonstrate TAPSβ and CAPS in genome-wide sequencing and long-read sequencing. We added one sentence in the discussion to reflect this.

2. Does hTet2 have any preference for CpG or non-CpG sites?

Yes, hTet2 has the preference for CpG sites according to previous reports ([Cell, 2013, 1545–1555](https://www.cell.com/fulltext/S0092-8674(13)01469-4); [Nature, 2015, 118–122](https://www.nature.com/articles/nature15713)). To better quantify the difference, we have added results of Illumina-TAPS with hTet2. Please see the above reply to review #1, which showed hTet2 has 97.3% conversion on CpG methylation and 74.0% conversion on non-CpG methylation.

3. The authors demonstrated that 50 ng genomic DNA is sufficient for the presented method. Have they tried any amount lower than 50 ng?

The genomic DNA input amount required for lrTAPS depends on the copy number of the target amplicon, PCR efficiency etc. In the presented method, we are aiming at demonstrating general applications of lrTAPS and 50 ng genomic DNA worked for all target amplicons. We tried lower than 50 ng, such as 10 ng or 1 ng, which did not yield enough PCR products within the same PCR cycles while PCR artefacts arise when more PCR cycles were added. We are working to address this problem by optimization of PCR primers and using nested PCR which could be applied on low-input clinical samples.
